# Adrenergic and Glucocorticoid Receptors in the Pulmonary Health Effects of Air Pollution

**DOI:** 10.3390/toxics9060132

**Published:** 2021-06-04

**Authors:** Myles X. Hodge, Andres R. Henriquez, Urmila P. Kodavanti

**Affiliations:** 1 Oak Ridge Institute for Science and Education Research Participation Program, U.S. Environmental Protection Agency, Research Triangle Park, NC 27711, USA; hodge.myles@epa.gov (M.X.H.); andhencor@gmail.com (A.R.H.); 2 Public Health and Integrated Toxicology Division, Center for Public Health and Environmental Assessment, U.S. Environmental Protection Agency, Research Triangle Park, NC 27711, USA

**Keywords:** adrenergic receptors, glucocorticoid receptors, air pollution, ozone, lung injury, inflammation

## Abstract

Adrenergic receptors (ARs) and glucocorticoid receptors (GRs) are activated by circulating catecholamines and glucocorticoids, respectively. These receptors regulate the homeostasis of physiological processes with specificity via multiple receptor subtypes, wide tissue-specific distribution, and interactions with other receptors and signaling processes. Based on their physiological roles, ARs and GRs are widely manipulated therapeutically for chronic diseases. Although these receptors play key roles in inflammatory and cellular homeostatic processes, little research has addressed their involvement in the health effects of air pollution. We have recently demonstrated that ozone, a prototypic air pollutant, mediates pulmonary and systemic effects through the activation of these receptors. A single exposure to ozone induces the sympathetic–adrenal–medullary and hypothalamic–pituitary–adrenal axes, resulting in the release of epinephrine and corticosterone into the circulation. These hormones act as ligands for ARs and GRs. The roles of beta AR (βARs) and GRs in ozone-induced pulmonary injury and inflammation were confirmed in a number of studies using interventional approaches. Accordingly, the activation status of ARs and GRs is critical in mediating the health effects of inhaled irritants. In this paper, we review the cellular distribution and functions of ARs and GRs, their lung-specific localization, and their involvement in ozone-induced health effects, in order to capture attention for future research.

## 1. Introduction

Circulating stress hormones are ligands for adrenergic receptors (ARs) and glucocorticoid receptors (GRs), ubiquitously distributed in the body; they are essential for responding to stress signals, orchestrating stress responses, and maintaining the homeostatic physiological function of major organ systems. In the lungs, these receptors have key roles in bronchoconstriction, microvascular contractility, maintaining immune surveillance, and alveolar patency [1,2]. Thus, it is conceivable that air pollution exposure, which causes irritation, alteration in breathing, and subsequent inflammation, directly or indirectly involves the activity of these receptors. However, until recently, only a few studies have examined the role of ARs or GRs in mediating the health effects of air pollution [3,4,5,6,7]. Some studies have assessed the contributions of ARs to pulmonary inflammation [8], whereas others have examined their role in the cardiovascular health effects of air pollutants [9]. Likewise, the contribution of GRs has also been examined in a limited number of air pollution studies [10]. This is despite the extensive therapeutic manipulation of these receptors for major chronic lung diseases, such as asthma and chronic obstructive pulmonary disease (COPD) [11]. For both diseases, the first-line therapy involves the use of beta-2 AR (β_2_AR) agonists as bronchodilators and steroidal GR agonists as immunosuppressants, which function to inhibit lung inflammation [12,13]. As AR and GR agonists have lifesaving therapeutic implications for lung diseases, their involvement in orchestrating and resolving lung injury and inflammation after air pollution exposure warrants further attention.

**Figure 1:**
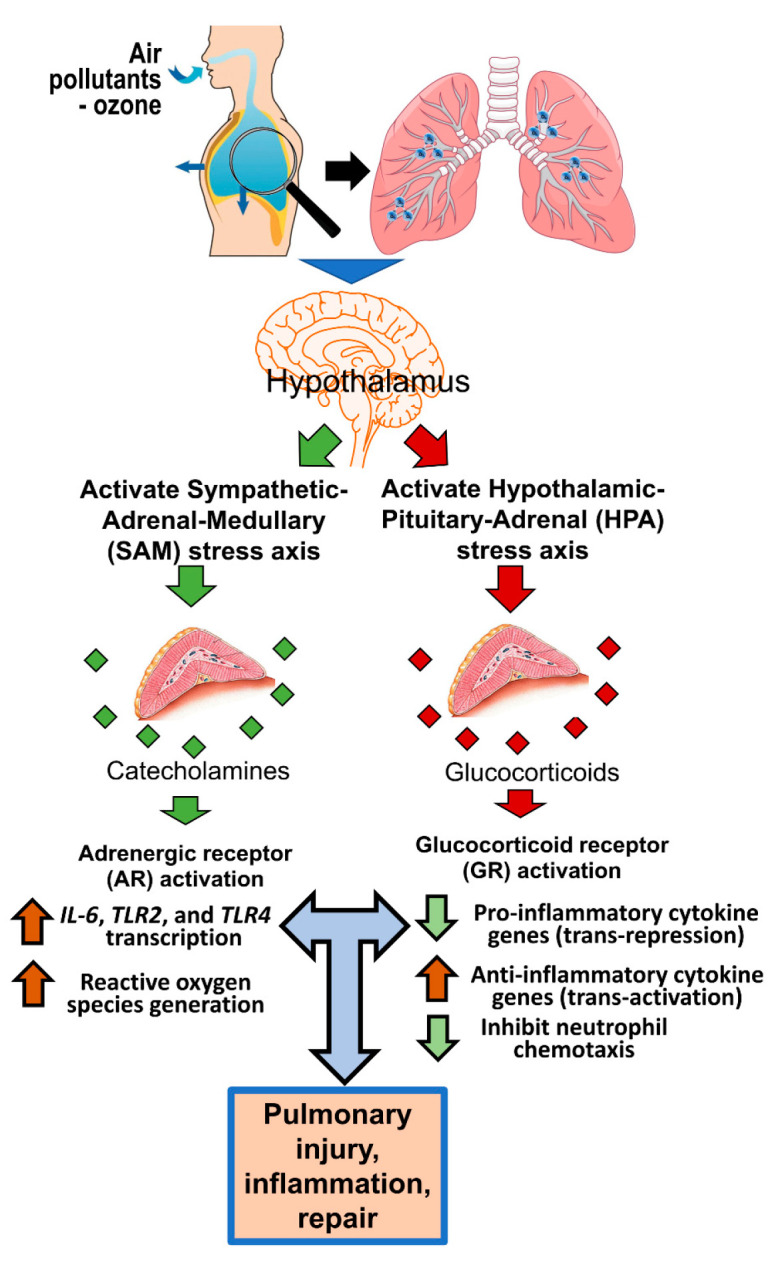
A flow chart of how air pollutant exposure through the neuroendocrine pathways activates adrenergic (ARs) and glucocorticoid receptors (GRs) and influences pulmonary response. Upon inhalation, air pollutants likely activate autonomic sensory nerves, which relay stress signals to the hypothalamus though the brainstem. This stimulates the hypothalamus to induce changes in the neuroendocrine pathways, including the activation of SAM and HPA axes, which results in release of catecholamines, such as epinephrine, and cortisol/corticosterone, into circulation. These hormones mediate their effects through widely distributed receptors for catecholamines (ARs) and glucocorticoids (GRs). These receptors—in addition to mediating homeostatic changes in physiological processes, and diurnal variations—respond to air pollution stress and direct bodily immune and metabolic responses at the site of injury. These processes result in a local inflammatory response that is governed by multiple organs, including the brain. *IL-6*: interleukin 6; *TLR2*: toll-like receptor 2; *TLR4*: toll-like receptor 4.

Our recent studies have examined the roles of these receptors in mediating ozone-induced lung injury and inflammation where ozone has been used as a prototypic air pollutant. We have shown that the activation of these receptors is necessary in mediating pulmonary injury and inflammation after ozone exposure (Figure 1) [4,5,6,7,14,15], and believe that they will have much broader implications for the health effects of air pollution. The goal of this paper is to discuss the critical contribution of ARs and GRs in the acute pulmonary effects of ozone, and we propose integrating the potential roles of these receptors in future studies involving other air pollutants. First, we will provide a general perspective on the functions and distribution of AR and GR subtypes, their involvement in homeostasis, and cellular signaling resulting from changes in circulating ligands for these receptors. Then, we will focus on the distribution of ARs and GRs in the lungs, and their relevance in chronic cardiopulmonary diseases. Based on the roles of ARs and GRs in the lungs, we will explain how ozone exposure leads to increased activity of these receptors. We will discuss how ARs’ and GRs’ cellular signaling may be involved in modulating pulmonary injury, vascular leakage, inflammation and even circadian changes through their activation. Finally, we will emphasize the importance of integrating the functions of these receptors in future air pollution studies examining mechanisms of pulmonary injury, inflammation, therapeutic interventions, and a potential link to altered diurnal rhythmicity.

## 2. ARs and GRs, Their Subtypes, and Roles in Homeostatic Functions

Adrenal-derived catecholamines (epinephrine and norepinephrine) and glucocorticoids that are released in the blood in response to stress maintain vital homeostatic functions through binding to their receptors—ARs and GRs, respectively [16,17,18]. The stress signal, perceived or physiological, conveyed through the autonomic sensory nerves or generated within the central nervous system (CNS), leads to hypothalamic sympathetic activation, which initiates the body’s response through the activation of the neuroendocrine system [19]. The activation of sympathetic nerves with wide cellular distribution among different organs releases norepinephrine (NE) at nerve terminals, which, in a paracrine manner, bind to ARs on effector cells in order to mediate immediate changes in cellular function in response to stress [20]. The other important function of sympathetic nerves, which innervate the adrenal medulla, is to mediate the synthesis and release of epinephrine and norepinephrine into circulation through the sympathetic–adrenal–medullary (SAM) axis (Figure 1) [21]. On sympathetic activation, the adrenal medulla releases catecholamines—endogenous ligands with differing affinities for AR subtypes. The activation of the sympathetic nervous system in response to stress is also associated with hypothalamic release of corticotropin-releasing hormone through portal circulation to the anterior pituitary, which then stimulates the synthesis and release of adrenocorticotropic hormone into systemic circulation [22]. Once released, this hormone stimulates the synthesis and release of corticosteroids (referred as cortisol in humans and corticosterone in rodents) and mineralocorticoids into circulation through the hypothalamic–pituitary–adrenal (HPA) axis. Cortisol and mineralocorticoids released into circulation then bind to GRs and mediate cellular responses to stress (Figure 1) [23,24]. Impairment of the SAM and HPA axes has been linked to a wide array of neuropsychiatric conditions, and even chronic peripheral diseases [16,17].

Through varied subtypes, cell- and tissue-specific distribution, and substrate specificities, as well as transcriptional and translational modifications, ARs and GRs maintain diurnal and stress-induced cardiovascular, respiratory, metabolic, and immune functions [1,25]. The diversity of the receptor subtypes, the plasticity of their function, and their cooperativity with other signaling regulators, along with variations in their distribution density at organ and cellular levels, enable precise and coordinated response tailored to a stressor in a cell- and organ-specific manner. The oscillatory and diurnal pattern of release of these hormones enables temporal regulation of normal cellular physiological functions, whereas stress-induced increases are critical to cellular changes that enable the cellular response to be phenotypically expressed in a reversible manner [26,27].

The two major types of AR, alpha and beta (αARs and βARs), each having several different subtypes, mediate the peripheral and central effects of catecholamines [28]. α_1_ARs, with affinity for both epinephrine and norepinephrine, are widely distributed in smooth muscles, where they mediate contraction [29]. α_2_ARs, on the other hand, are mostly presynaptic for adrenergic and cholinergic nerve terminals, and they counteract the sympathetic effects of smooth muscle contraction (Table 1) [29]. β_1_ARs, with similar affinity between epinephrine and norepinephrine, are distributed predominantly in the heart and kidney muscles, where they induce muscle contraction to increase heart rate and renin release in glomerular cells (reviewed in [30]). β_2_ARs are widely distributed in the respiratory, vascular, and uterine smooth muscles, where they increase relaxation, regulate fluid balance, and influence inflammatory responses, and in the liver they mediate glucose release. β_3_ARs are predominant in adipose tissue and the bladder wall, where they increase adipose lipolysis and relax the bladder muscle, respectively [31].

GR-α (a predominant GR and, thus, generally referred to as GR), with various splice forms, is universally distributed in all cells/organs, and is activated by cellular glucocorticoids to induce tissue-specific metabolic and immune changes through genomic and nongenomic mechanisms [25,34]. Less widely distributed GR-β is located in the nucleus, and antagonizes the transcriptional activity of GR-α through varied mechanisms [25]. GR activation suppresses the immune response by directly inhibiting the gene expression of proinflammatory mediators [31]. In some instances, glucocorticoid-bound GRs can also act through nongenomic mechanisms to affect cellular function [35]. Precise regulation of the downstream effects of GR activation is achieved through various posttranscriptional alterations, regulatory processes, and cooperativity with other transcription factors within the nucleus.

## 3. Cellular Signaling through Activation of ARs

**Figure 2:**
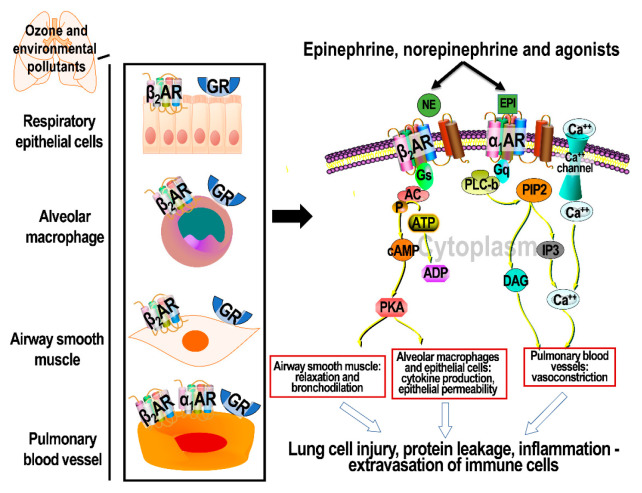
Schematic showing the cellular effects of activating AR subtypes in the lung. The left panel shows the distribution of α_1_AR, β_2_AR and GR in lung cells. The right panel shows cell signaling through α_1_AR and β_2_AR. β_2_AR signaling involves cAMP-mediated activation of PKA through phosphorylation, and effects on transcription factors that mediate the expression of genes regulating bronchodilation, inflammation, and epithelial transport. α_1_AR signaling, on the other hand, leads to increases in intracellular free calcium though the activation of phospholipase C and diacylglycerol, where the activation of PKC causes pulmonary vasoconstriction. β_2_AR: beta 2 adrenergic receptors; α_1_AR: alpha 1 adrenergic receptors; ATP: adenosine triphosphate; cAMP: cyclic adenosine monophosphate; PKA: protein kinase A.

Epinephrine and norepinephrine released into circulation from adrenal glands and nerve terminals activate ARs, which are G-protein-coupled receptors (GPCRs) to induce cellular changes in tissues, including lung (Figure 2) [36,37]. Catecholamine binding to ARs mediates rapid muscle contraction or relaxation [38], increases heart rate [39], and stimulates adipose lipolysis [40]. The activation of ARs through these ligands initiates a cascade of events involving distinct G proteins subsequently producing second messengers [41,42]. Nine subtypes of αARs and βARs (α_1_a, α_1_b, α_1_c, α_2_a, α_2_b, α_2_c, β_1_, β_2_, and β_3_) have specific ligand-binding properties, and involve different but coordinated regulatory mechanisms [43]. α_1_ARs are G-protein-aq (G_aq_)-coupled, and phosphorylate protein kinase C, which mediates the production of inositol triphosphate (IP3) and diacylglycerol (Figure 2). These increases in second messengers enhance the intracellular release of free calcium, causing further activation of protein kinase C, which is involved in multiple signaling mechanisms [29]. α_2_ARs, when bound to ligands, couple with G protein ai (G_ai_), and exert autoinhibitory effects by decreasing protein kinase A (PKA) and inhibiting the production of cyclic AMP (cAMP) by adenylate cyclase and, thus, counteracting the effects of α_1_AR [29].

On the other hand, βARs are G-protein-as (G_as_)-coupled, and involve the activation of protein kinase A (PKA) through stimulation of adenylate cyclase and cAMP production. The binding of hormone ligands to β_1_AR leads to the production of a number of second messengers that are involved in the downstream activation of transcription factors. cAMP-dependent protein kinase A phosphorylates calcium channels, leading to increased concentrations of intracellular calcium, facilitating contraction of the myosin light chain and, thus, the contractility of muscle cells [30]. The activity of β_2_AR-mediated PKA signaling in airway smooth muscle cells involves other proteins, such as phospholipase C, myosin light-chain kinase (MLCK), IP3, calcium channels, and heat shock protein 20, which, when phosphorylated, inhibit signaling that leads to smooth muscle contraction (Figure 2) (reviewed in [33]). β_2_AR may also coordinate with reduced nicotinamide adenine dinucleotide phosphate (NADPH) oxidase through PKA and β-arrestin to mediate oxidative stress [44].The activation of GPCR G_as_ by ligand binding to βARs leads to its phosphorylation by kinases, facilitating binding of one of the four β-arrestins to the complex [45]. A cascade of events follows, leading to autoregulation of further activation, preparation of the receptor complex for internalization, and activation of other signaling pathways, such as extracellular signal-regulated kinases (ERKs) [46]. βARs also complex with G protein bg (G_bg_) to induce intracellular signaling and mediate functions of ion channels, as well as the activation of phospholipase C and G protein receptor kinase [47]. In addition to these canonical pathways that mediate signaling to induce second messenger activation, βARs also mediate signaling that does not involve G proteins and subsequent cAMP production. For example, β_2_AR can activate the glycogen synthase kinase 3b signaling pathway, which involves serine/threonine protein kinase (AKT) [48], while β_1_AR can also mediate signaling through other kinases, including mitogen-activated protein kinase (MAPK) and stress-activated protein kinase (SAPK), to induce transcriptional changes [49]. The activation of GPCRs and production of second messengers are regulated temporally based on diurnal cycle, and spatially to mediate diverse cellular changes [50]. Moreover, there is an oscillatory pattern of changes in AR-mediated second messenger production, leading to differential amplitude and frequency of their actions, which may program temporally different downstream responses based on diurnal cycle [51].

## 4. Cellular Signaling through Activation of GRs

**Figure 3:**
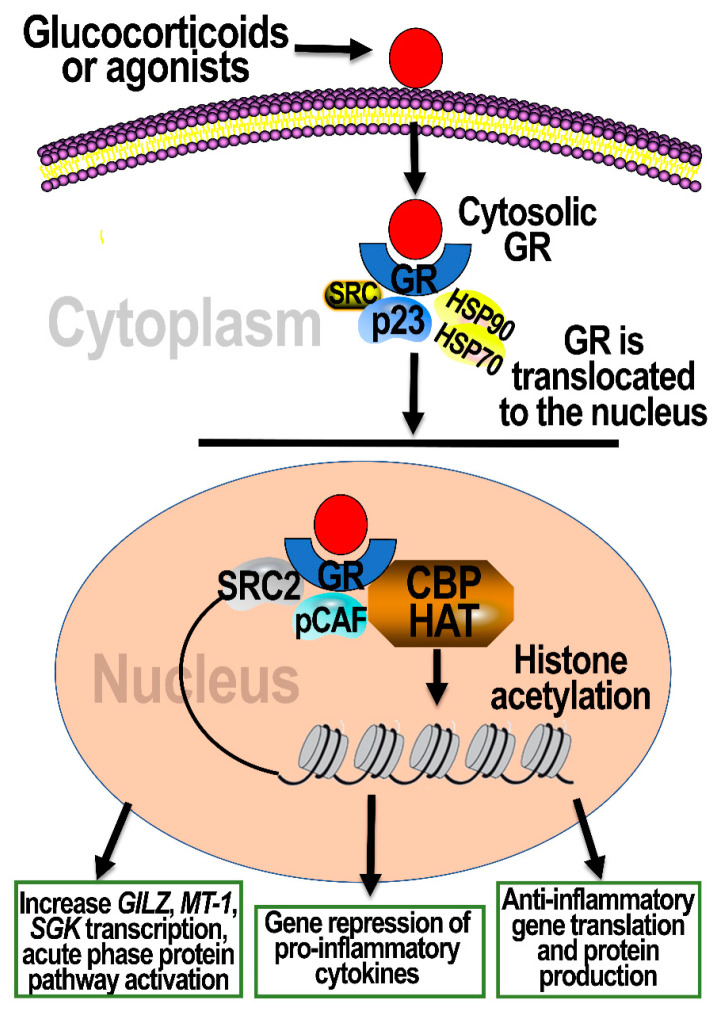
A schematic of lung cellular effects from activating GRs. Lipophilic glucocorticoids enter cells freely. Upon entering cells, glucocorticoids bind to GRs, which exist in the cytoplasm complexed with heat shock proteins 70 and 90 (Hsp70 and Hsp90), p23, and other proteins, such as steroid receptor coactivator (SRC). Upon binding to glucocorticoids, other proteins are recruited in the complex, preparing it for nuclear translocation. Once in the nucleus, GRs recruit P300/CBP-associated factor (pCAF), CREB-binding protein (CBP), and histone acetyltransferase (HAT), allowing complex to modify the chromatin framework and bind to glucocorticoid response elements (GREs) in promotor sequences of DNA. This results in transactivation or transrepression, leading to activation or inhibition of gene transcription. This is achieved through the direct binding of the GR complex to GREs and/or its interaction with other transcription factors (some details are not given in the figure for simplicity). Through their transcriptional regulation of gene expression, GRs change the expression of genes involved in inflammation, acute-phase response, and anti-inflammatory mechanisms. *GILZ*: glucocorticoid-induced leucine zipper; *MT-1*: metallothionein-1; *SGK*: serine/threonine-protein kinase.

Increases in circulating free lipophilic glucocorticoids lead to their cellular entry, binding to GRs and mediating a series of events leading to their nuclear translocation, binding to gene sequences at many different sites, and influencing the expression of a major pool of genes (Figure 3). The bioavailability of intracellular glucocorticoids is regulated by 11β-hydroxysteroid dehydrogenase 1, which converts glucocorticoids to cortisone, an inactive form, whereas 11β-hydroxysteroid dehydrogenase 2 converts cortisone to corticosterone or cortisol [52]. The spatial and cellular distribution of these enzymes can control the availability of glucocorticoids to bind to GRs. On binding to GRs, glucocorticoids at physiological levels regulate immune and metabolic homeostasis; however, under acute stress, glucocorticoids also can act to increase proinflammatory mediators when concentrations reach critical levels [53,54]. As reviewed by Oakley and Cidlowski [25], GRs constitute a major class of nuclear transcription factors, and are estimated to regulate about 10–20% of the human genome.

Human GRs are encoded by *NR3C1*, where 13 variants of axon 1 harbor binding sites for numerous transcription factors, including binding for GRs themselves, which enables tight regulation of their own expression. Axon 1 of *NR3C1* is also regulated by epigenetic modifications (reviewed in [13]). By transcriptionally regulating the expression of thousands of genes (transactivation and transrepression), the specificity of GRs is achieved by their different splice variants and context-specific regulation. Cytosolic GRs, in the absence of glucocorticoids, exist as monomers complexed with other chaperone proteins, aiding to their maturation for transcriptional activity. On binding to glucocorticoids, GRs complexed with a number of proteins that are replaced by FK506-binding protein 51 (FKBP51) and p23 chaperone protein, preparing complexes for nuclear translocation and DNA binding [13]. In addition to binding of GR homodimers to palindromic sequences on DNA (glucocorticoid response elements; GREs), GR complexes can also be exported back to the cytoplasm as another regulatory control of its transcriptional activity [55]. GR homodimer binding to GREs directly activates transcription of genes (transactivation) or silencing of transcription (reviewed in [13]). GRs can also partner with other transcription factors to modulate changes in gene transcription, including activator protein 1 (AP-1), nuclear factor kappa B (NF-κB), and signal transducers and activators of transcription (STAT). By transactivation and by interacting with AP-1 and NF-κB, GRs increase expression of proinflammatory genes. Through interaction of GREs with the STAT family of transcription factors, GRs enhance the transcriptional activity of genes regulated by this transcription factor (reviewed in [25]).

The effects of GR activation on gene expression are diverse, and regulated by complex cytoplasmic and nuclear signaling processes, which are central to producing the needed proteins involved in orchestrating dynamic cellular physiological response to stress. GRs have been shown to interact with NF-κB in the cytosol of rat liver cells through nongenomic mechanisms. To suppress the nuclear translocation of NF-κB, cytosolic GRs, even without ligand binding, physically interact with p65/p50 and IκB subunits [56,57]. Inflammatory stimuli upregulate MAPK-associated pathways, activating stress-responsive proteins, glucocorticoid-induced leucine zipper (GILZ, also known as TSC22d3), MAPK phosphatase-1, and annexin-1 [56]. Moreover, MAPK phosphatase-1 and annexin-1 suppress the MAPK pathway to decrease the inflammatory response [58,59]. GR binding to glucocorticoids has been shown to modulate other cellular processes that are not regulated by genomic mechanisms; for example, GRs induce changes in membrane configuration, alter MAPK signaling through the GR-binding proteins, and regulate the transcription of mitochondrial genes [60].

Plasticity in GR-mediated biological processes is complex and influenced by multiple regulatory impacts on its activity [17]. Posttranslational modifications of GR, including phosphorylation, ubiquitination, and acetylation, can also modulate GR activity. In addition, a single base pair change in the GRE binding consensus DNA sequences can change the binding capacity of GRs [61]. The accessibility of GRs to specific GREs is also regulated by the chromatin landscape and DNA-binding proteins in given cell types, where GREs that are easily accessible to GRs are thought to be occupied at low concentrations of glucocorticoids, giving another option for concentration-dependent differences in the types of genes being activated. These multiple controls on the transcriptional activity of GRs enable plasticity in regulating homeostatic physiological processes (reviewed in [13,25,62]).

## 5. Distribution of AR and GR Subtypes in the Lungs

Conducting airways, parenchyma, pulmonary vasculature, and epithelial cells selectively express AR and GR subtypes that are essential in maintaining the homeostatic function of the lungs [1]. α_1_ARs are expressed in pulmonary and vascular smooth muscle cells, and mediate vasoconstriction [63]. Of all ARs, βARs are distributed more abundantly and widely in various cells of the lungs. They are expressed in vessel walls, airway smooth muscles, submucosal glands, and on distal airways and alveolar walls [1,64]. β_2_ARs make up approximately 70% of all pulmonary βARs [65]. β_2_ARs are specifically present in airway, vascular smooth muscle, and epithelial cells, whereas β_1_ARs are primarily localized in alveolar walls and submucosal glands. In the alveolar walls, β_1_ARs are more abundant when compared with β_2_ARs [1]. β_2_ARs have been shown to play a role in alveolar fluid clearance, as well as immune surveillance [65,66]. When activated by its ligands, such as epinephrine or other beta-agonist drugs, β_2_ARs cause an increase in intracellular cAMP and calcium channel activation to cause smooth muscle relaxation and bronchodilation (Figure 2) [67]. In the lungs, capsaicin sensory nerves and mast cells also express β_2_ARs. Their expression in other immune cells in the lungs is relatively less abundant [1]. Although other immune cells in the lungs express low levels of β_2_ARs, they are also expressed in pulmonary cells, predominantly in airways and endothelial cells [68,69]. β_3_ARs present in pulmonary vascular smooth muscle are known to cause vasodilation of the pulmonary artery influencing cAMP-dependent pathway [69,70,71]. However, the underlying mechanisms by which each AR subtype modulates the pulmonary effects of air pollutants remain largely unelucidated.

GRs (GRαs) have much wider tissue distribution throughout the body, including the lungs. All structural and immune cells within the lungs express GRs, but likely with differential density and different coregulatory mechanisms to enable cell-specific effectiveness of glucocorticoids [1]. It has been suggested that the endothelial and pulmonary epithelial cells, which readily secret proinflammatory mediators, may express GRs more abundantly than other cell types, which have specific roles in maintaining homeostatic immune regulation at the air–liquid interphase [1]. In asthmatic lungs when compared to healthy, GRs are distributed much more widely among smooth muscle cells, fibroblasts, and macrophages, and may play a role in controlling overly activated inflammatory responses [72]. Because GRs are also distributed widely in other organ systems, inhaled steroids are used to reduce the local pulmonary inflammatory response without causing systemic effects in patients with asthma and COPD. The majority of GR subtypes in tissues are GRαs. GRβs, which antagonize the effects of GRαs, are present in relatively low levels in tissues, and are sparsely distributed in the lungs. It has been shown that steroid-resistant asthma patients have increased expression of GRβs [73].

ARs and GRs are prime therapeutic targets for treating pulmonary and cardiovascular diseases. β_2_AR agonists are used in patients with COPD and asthma, whereas antagonists of β_1_AR are used for hypertension and more advanced cardiac complications, such as heart failure [74,75,76]. The therapeutic use of GR agonists can greatly reduce the inflammatory response, both in the lungs and systemically [77]. Generally, the combination of β_2_AR agonists and glucocorticoids is prescribed to asthma and COPD patients in order to promote bronchodilation and reduce inflammation [78]. Because irritant pollutants induce increases in circulating endogenous epinephrine and glucocorticoids, which function as ligands for ARs and GRs, respectively, the understanding of AR and GR signaling in pulmonary and cardiovascular diseases can aid in determining their roles in modulating pulmonary and cardiovascular responses to inhaled air pollutants.

## 6. ARs and GRs in Air-Pollutant-Induced Lung Injury and Inflammation

When inhaled, physiochemically diverse pollutants produce local cellular changes and activate cell signaling pathways that promote cell injury, proinflammatory cytokine release, and oxidative cell changes, leading to immune cell extravasation to the pulmonary tissue [79,80]. Although local pulmonary cellular changes have been characterized extensively with acute exposure to different pollutants, the mechanisms by which the immune response is activated and immune cells are recruited from lymphoid organs, matured, and extravasated to the site of injury are not well understood. Our recent studies have shown that the adrenergic and glucocorticoid pathways are involved in pulmonary vascular leakage and inflammatory response induced by irritant pollutants, such as ozone [14,81,82]. In light of recent research into the marked effects of air pollutants on the brain [7,82,83,84], and the involvement of the neuroendocrine system with increased adrenal-derived stress hormones [85], it is conceivable that AR and GR activation are involved in the health effects of air pollution. Understanding the roles of these receptors may explain how a pulmonary inflammatory response is generated after exposure to air pollutants, why there is tolerance or adaptation to this initial pulmonary response, how individuals with psychosocial disorders and altered neuroendocrine regulation may be more susceptible, and what systemic mediators are critical for initial injury and inflammation.

### 6.1. Air Pollution Studies Implicating the Role of ARs

Epidemiological studies have highlighted that polymorphisms in β_2_AR, especially Arg16, increase the risk of airway hyperresponsiveness in asthma, decrease forced expiratory volume in one second (FEV_1_), and impair overall lung function [86,87]. Particulate matter (PM) exposure in dogs induces peripheral vascular resistance via αAR activation. This effect is attenuated with the αAR antagonist, prazosin [88]. Moreover, a number of epidemiological studies have implicated increases in stress hormones and exposure to air pollutants [89,90,91], which may involve their effects on ARs, GRs, and downstream cellular changes (Table 2).

The contributions of β_2_AR signaling to cigarette-smoke-, lipopolysaccharide (LPS)-, and other pollutant-induced lung injury and inflammation have been examined in few experimental studies. β_2_AR signaling through GPCR-kinase-mediated phosphorylation and binding to β-arrestin can influence inflammatory cell signaling. Given the contribution of β-arrestin 2 activation to inhibiting autophagy via the adenosine monophosphate-activated kinase (AMPK)/mammalian target of rapamycin (mTOR) pathway, and the suppression of inflammatory cytokine production in human bronchial epithelial cells (BEAS-2B) exposed to cigarette smoke [92], it will be important to determine how the activation of β_2_AR after air pollution exposure may contribute to inflammation in the lungs through β-arrestin. In human blood monocytes, activation of βAR subtypes by isoproterenol repressed the LPS-induced secretion of inflammatory cytokines, such as TNF-α and IL-6 [100]. In the same manner, immunosuppression through inhibiting the NF-κB pathway occurred in bone-marrow-derived macrophages with norepinephrine binding to ARs [93]. β_2_AR’s interaction with toll-like receptors has been shown to cause immunosuppression through upregulation of anti-inflammatory gene expression [101]. The inhibitory effects of βAR agonists on immune response have been shown to occur through the negative regulation of type 2 innate lymphoid cells [102]; however, when the β_2_AR transgene was overexpressed in gene knockout or in wild-type mice, the proinflammatory phenotype was exacerbated [103]. Thus, although the evidence for β_2_AR’s involvement in immunosuppression is substantial [104], the recent evidence suggests its contribution to promoting innate inflammatory responses through increased IL-6 production [8]. Experimentally, it has been shown that acute exposure to air pollutants, such as ozone and acrolein, activates the sympathetic nervous system, causing local and systemic secretion of catecholamines [8,14,94,105]. The activation of β_2_AR by agonists potentiates pulmonary inflammatory responses through exacerbating macrophage release of IL-6 [8]. The activation of β_2_AR in alveolar macrophages by ambient PM was demonstrated to induce the release of mitochondrial reactive oxygen species and phosphorylation of the cAMP-response-element-binding protein (CREB) to increase *IL-6* transcription [8]. Recently, Richie et al. [106] have also shown that increased IL-6 production by β_2_AR agonists in cells infected with respiratory syncytial virus involved the cAMP response element (CRE). Moreover, overexpression of *α_2_bAR* (*Adra2b*) in transgenic mice resulted in increased gene expression of *Il-6*, *Tlr2*, and *Tlr4*, enhancing inflammation in the brain following PM exposure [95].

The involvement of ARs has been shown in other air pollution studies examining cardiovascular effects. Increases in intracellular free calcium in endothelial cells after exposure to diesel exhaust particles were shown to involve βARs [3]. In a rat model of acute myocardial infarction, exposure to particulate matter resulted in exacerbation of injury, while treatment with metoprolol, a β_1_AR-specific blocker, reduced the effect [9]. The vasoconstriction response observed in isolated microvessels from PM-exposed rats was inhibited by αAR blockade [96]. Thus, some studies have emphasized the role of different AR subtypes in mediating pulmonary and cardiovascular effects of air pollutants; however, the cellular mechanisms, and how pollutant exposure results in increased circulating ligands for ARs, are just beginning to emerge. Although the role of sympathetic activation in mediating cardiovascular effects has been established [107,108], and epidemiological studies have shown associations between circulating catecholamines and air pollution [91], the mechanisms of central regulation mediating the release of catecholamines responsible for activating ARs have yet to be demonstrated in ambient PM studies.

### 6.2. Air Pollution Studies Implicating the Role of GRs

As a few studies have implicated the involvement of ARs in mediating inflammatory responses in the lungs after air pollution exposure, even fewer studies have examined the role of GRs (Table 2). This is despite significant evidence that air pollution may induce resistance to inhaled glucocorticoids in patients with asthma and COPD [109]. Jia et al. [97] reported that the levels of cortisol were increased in humans exposed to air pollutants, and this effect was recapitulated in mice after exposure to heavy air pollution, and was associated with hippocampal inflammation as well as behavioral alterations. These studies imply that glucocorticoids are likely involved, and mediate their effects centrally through the activation of GRs. The endocrine-disrupting effects of metals were examined in vitro using different cell lines, including mouse macrophage cells using reporter luciferase assay for GR activation [98]. This study showed that GR activity was inhibited by selected metals. The effects of PM exposure on multiple organs, including the lungs, were linked to increased glucocorticoid activity in rats [99]. Although these studies show the involvement of GRs in mediating the acute effects of PM, it is not known how these receptors may be activated, or what coregulatory mechanisms are involved in mediating pulmonary vascular leakage and inflammation secondary to peripheral changes in immune cells. The understanding of the mechanisms by which air pollutants stimulate the SAM and HPA stress axes, and their contribution to individual susceptibility variations though GRs, could inform mitigation and therapeutic strategies.

## 7. ARs and GRs in Ozone-Induced Lung Injury and Inflammation

The strongest evidence for the role of catecholamines and glucocorticoid activation of ARs and GRs in mediating pulmonary injury and inflammation comes from our acute ozone inhalation studies [5,6,7]. Although the mechanisms of ozone-induced lung injury and inflammation are well characterized, the evidence that ozone exposure leads to increases in epinephrine and corticosterone in rats [14,105] and cortisol in humans [110] suggests that the receptors of these stress hormones are likely involved in mediating ozone’s effects [111]. A number of our recent studies have demonstrated the contribution of ARs and GRs to mediating ozone-induced lung injury and inflammation (Table 3). Ozone was used as a prototypic air pollutant known to induce oxidant injury in the lungs [112], and is not likely to translocate systemically, allowing us to test the contribution of the neuroendocrine system without direct effects in the periphery [80]. Below is an account of a series of studies that emphasized the role of ARs and GRs in mediating ozone’s effects.

### 7.1. Adrenalectomy Inhibits Lung Injury and Inflammation Induced by Acute Ozone Exposure

We have shown that by eliminating the circulating ligands (catecholamines and glucocorticoids) for ARs and GRs via total bilateral adrenalectomy, the ozone-induced lung vascular leakage, injury, and inflammation were nearly eliminated, providing us with first-hand evidence of the contribution of circulating epinephrine and corticosterone and their receptor targets in mediating ozone’s acute effects in rats [113]. More importantly, when only the adrenal medulla was removed while keeping the cortex in place through adrenal bilateral demedullation, which diminished circulating epinephrine while only marginally affecting levels of corticosterone, there was also a reduction of ozone-induced lung injury and inflammation, suggesting that circulating epinephrine, which binds to ARs, was critical in mediating pulmonary effects [113]. These findings were further supported by the observation that global transcriptional changes in the lungs induced by ozone in normal rats (over 2000 genes changed) were reduced by over fivefold in animals with adrenal demedullation or total adrenalectomy [114]. It is noteworthy that ozone-induced lung injury and inflammation are noted only after the first 2 days of ozone exposure; however, these effects of ozone are reversible despite daily repeated exposure for 3 or more consecutive days, suggesting adaptation.

Adrenalectomy, in addition to eliminating epinephrine and corticosterone from circulation, also depletes mineralocorticoids important in the osmotic balance of salt and water, and vascular function, thus removing the influence of endogenous ligands for ARs and GRs, as well as mineralocorticoid receptors. To assess the precise contributions of epinephrine and corticosterone, we performed a gain-of-function experiment by treating adrenalectomized rats with β_2_AR plus GR agonists, and assessed ozone-induced lung injury, systemic and pulmonary inflammation, and cytokine gene expression [4]. In this study, β_2_AR agonist was selected based on their enriched distribution in the lungs and functional significance [1]. The reduction of ozone-induced pulmonary and systemic effects by adrenalectomy, and the restoration or even exacerbation of this effect in adrenalectomized rats by treatment with a combination of β_2_AR and GR agonists, suggest that ozone’s effects on the lungs are mediated by the activation of these receptors, and not because of the effects on circulating mineralocorticoids.

### 7.2. βAR and GR Activation Contribute to Ozone-Induced Lung Inflammation

We have also assessed the independent roles of βARs and GRs in ozone-induced inflammation by inhibiting GRs while pharmacologically activating βARs, or by inhibiting βARs while pharmacologically activating GRs, prior to exposing rats to ozone [15]. These experiments demonstrated that the effects of βARs and GRs are manifested independently after ozone exposure in animals. The involvement of ARs and GRs in ozone-induced lung injury and inflammation suggests a potential interaction of the therapeutics used for asthma and COPD, which work through the same receptor system. As ozone increases circulating epinephrine and corticosterone [14,105], it can be presumed that those taking combination treatment of β_2_AR and GR agonists will have exacerbated inflammatory and pulmonary functional outcomes of the disease upon air pollution exposure. Children with asthma using maintenance medication have greater pulmonary inflammatory responses to ozone than those not receiving medication [116], further supporting the contribution of AR and GR activation in adverse pulmonary and systemic health outcomes.

Glucocorticoids exert their anti-inflammatory effects by reducing the transcription of proinflammatory cytokines and influencing the egress and margination of granulocytes and lymphocytes from lymphoid organs [25]. The findings that ozone-induced depletion of circulating T and B lymphocytes was reversed by adrenalectomy in rats, and that the ozone’s effect was regained by pretreating adrenalectomized rats with dexamethasone (GR agonist) plus clenbuterol (β_2_AR agonist), support the role of βARs and GRs in mediating systemic immune effects and pulmonary inflammation [4]. Our recent study examined the kinetics of stress hormone release together with changes in the pool of various immune cells in the circulation, as well as changes in circulating cytokines during a 4-h ozone exposure [85]. This study clearly demonstrated that the depletion of circulating granulocytes, monocytes, T helper cells, cytotoxic T cells, and B cells, occurred after increases in circulating epinephrine and corticosterone within 1 h of ozone exposure. The increases in circulating stress hormones, and subsequent increases in the expression of glucocorticoid-responsive genes in the lungs, along with the depletion of circulating immune cells, were noted, with only minimal changes in the circulating cytokines. Therefore, these data suggest that the stress-hormone-mediated activation of ARs and GRs likely leads to the pulmonary and systemic effects of ozone [85]. Ozone-induced upregulation of *Tsc22d3*, metallothionein 1 (*Mt-1*), and *Fkbp5* in the lungs following increases in stress hormone levels suggests the activation of GR-responsive genes by increased corticosterone [4,6,14,85,117].

To further assess the therapeutic relevance for those using dual therapy with agonists of β_2_AR and GRs, and how this may modulate ozone-induced lung injury and inflammation, we treated rats with β_2_AR and GR agonists, individually or in combination, at therapeutically relevant dose levels prior to ozone exposure. In this study, we demonstrated that ozone-induced lung injury and inflammation are highly exacerbated by treatment with the β_2_AR agonist clenbuterol, especially when given individually [6]. This exacerbation of ozone effects was dampened when combination therapy with clenbuterol plus dexamethasone was instituted. Likewise, a rhinovirus-induced increase in IL-6 was exacerbated by salmeterol, but this effect was dampened by coadministration of inhaled corticosteroids [118]. Since we used healthy animals in our studies, the implications of these findings need to be assessed carefully in asthmatic animal models. Nevertheless, because β_2_AR and GR agonists are used extensively in the treatment of asthma and COPD, the health implications of air pollution affecting the activity of these receptors, especially for individuals receiving these agonists, could be significant.

### 7.3. AR and GR Antagonists Inhibit Ozone-Induced Lung Inflammation

βARs are prominently distributed in the lungs, and therefore we further assessed their role in ozone-induced lung injury and inflammation [14,113]. We used propranolol, a nonspecific βAR antagonist, with or without the glucocorticoid antagonist mifepristone, to examine the contributions of individual receptor types in mediating lung vascular leakage and inflammation after ozone exposure. Our studies in rats show that inhibiting βARs using propranolol is associated with the inhibition of ozone-induced inflammation [5]. These data suggest that ozone-induced increase in epinephrine, which binds to β_2_AR, is associated with pulmonary inflammation; however, the precise mechanisms of cell- and organ-specific differences in βAR downstream signaling will need to be further assessed with air pollutant exposure [119]. The contribution of βAR signaling, and the differential involvement of β-arrestin activation, may underlie pollutant-specific differences in downstream signaling and inflammation.

GR manipulation during ozone exposure also provided insights into their role in pulmonary injury and inflammation. Ozone-induced increases in circulating corticosterone and subsequent lymphopenia, along with time-related depletion of granulocytes and M1 monocytes, suggest redistribution of circulating immune cells, likely marginating to the pulmonary vasculature, involving the activation of ARs and GRs [85]. Pretreatment of rats with the GR antagonist mifepristone, although reducing pulmonary protein leakage, was ineffective in reducing ozone-induced neutrophilic inflammation, suggesting that GR activation within the lungs was not involved in neutrophilic inflammation [5]. Interestingly, treatment with mifepristone reversed lymphopenia induced by ozone, implying that GR activation was necessary for systemic lymphopenia, and that glucocorticoids released after ozone exposure modulated the systemic immune response [5]. The combination of propranolol and mifepristone nearly eliminated ozone-induced lung vascular leakage, inflammatory cytokine induction, activation of glucocorticoid responsive genes, and the systemic immune response (lymphopenia), implicating βARs and GRs in ozone-induced inflammatory effects.

### 7.4. The Role of βARs in Ozone-Induced Lung Protein Leakage

It has been shown that vascular permeability increased by intravenous substance P injection in rats was blocked by the β_2_AR-specific agonist formoterol through its effect on endothelial gap junction reduction [120], contrary to what we observed with the β_2_AR-specific agonist clenbuterol, which was associated with increased protein leakage in air-exposed rats [6]. Moreover, the protein leakage induced by ozone exposure was highly exacerbated by clenbuterol pretreatment [4,6]. Because β_2_AR has high affinity for epinephrine relative to norepinephrine (Table 1), and because ozone specifically increased epinephrine levels in rats [14,105], it is likely that pulmonary β_2_AR might be involved in pulmonary vascular leakage; however, the contribution of cardiac β_1_AR and other associated hemodynamic changes cannot be ignored in mediating protein leakage.

Rats pretreated with propranolol alone demonstrated significantly reduced vascular leakage and lung inflammation after ozone exposure [5]. Increased circulating epinephrine can have marked effects on cardiac β_1_AR, pulmonary α_1_AR, and β_2_AR. Propranolol is a nonspecific βAR blocker, which can inhibit the activity of both β_1_AR and β_2_AR. Because of marked effects of epinephrine on β_1_AR in cardiac muscle contractility, and on β_2_AR in vascular and bronchial smooth muscle relaxation, an ozone-induced epinephrine increase may lead to hemodynamic changes in the low-pressure pulmonary vasculature. Moreover, due to parasympathetic dominance over sympathetic activation on the heart [121], even though an ozone-induced increase in epinephrine was apparent [14,105], cardiac depression in rats acutely exposed to ozone was associated with bradycardia [121]. This imbalance of sympathetic and parasympathetic influence might specifically exacerbate hemodynamic changes in the low-pressure pulmonary vasculature, leading to vascular protein leakage in the alveoli. Hypoxia-induced pulmonary injury in rats has been shown to involve catecholamines, such as epinephrine, and their action on αARs and βARs [122]. Both αARs and βARs have been implicated in pulmonary edema [123,124]. Based on the reversal of ozone-induced pulmonary edema by propranolol [5], it is likely that these receptors may also play an important role in the modulation of hemodynamic changes after air pollution exposure.

### 7.5. Circulating Ligands of ARs and GRs in Lung–Brain Communication

In order to better understand the contribution of the lung–brain axis through the activation of ARs and GRs to mediating ozone-induced pulmonary and systemic effects via increases in circulating epinephrine and corticosterone, we further examined circulating pituitary-derived hormones and gene expression changes in the stress-responsive regions of the brain, such as the hypothalamus and the brainstem, in rats with sham surgery and those with total bilateral adrenalectomy [7]. It has been previously shown that acute ozone exposure activates stress-responsive regions—such as the nucleus tractus solitarius—within the brain stem and the hypothalamus, where stress signals are processed [125]. In our study, a single ozone exposure was associated with marked gene expression changes in the brainstem and the hypothalamus, which reflected changes induced by hypoxia, inflammatory signaling, and steroidal as well as mTORC signaling, suggesting cellular homeostatic physiological alterations through adrenergic and steroidal signaling in both brain regions [7]. This was further supported by nearly 70% similarity in the genes upregulated by ozone in both of these brain regions. One of the important findings from this study was that no gene expression changes were noted in adrenalectomized rats exposed to air relative to sham rats, with severely depleted epinephrine and corticosterone [7], suggesting that stress signals through circulating AR and GR ligands were needed to induce changes in the rats’ brains. Moreover, virtually no gene expression changes occurred in ozone-exposed adrenalectomized rats, implying that in the absence of circulating epinephrine and corticosterone there were no cellular responses produced in either brain region, and that pulmonary AR and GR activation is necessary for ozone-induced gene expression changes to occur in the brain. Although adrenalectomy also markedly reduced lung gene expression [114], one could presume that the activation of ARs and GRs in the lung is critical for changes in the lungs and brain to occur after ozone exposure. Furthermore, communication between the lungs and the brain was needed in order to receive a stress signal in the brain from irritancy stress after ozone exposure. It is noteworthy that ARs and GRs have been implicated in stress adaptation and resiliency, and that the failure of normal functioning of these receptors has been linked not only to neurological ailments but also to peripheral chronic diseases [16,17,19]. Thus, the contribution of these receptors to air-pollutant-induced stress responses and subsequent pulmonary, neuronal, and peripheral effects, as well as adaptation, warrant further consideration.

## 8. Potential Interactive Roles of ARs and GRs in Inflammatory Mechanisms

Although research has uncovered that ARs and GRs influence inflammatory responses in the body and regulate physiological homeostatic processes, few studies have elucidated whether these receptors interact during the activation of molecular signaling pathways at the level of second messengers [126,127,128]. Given the diversity and the interconnectivity of AR/GPCR-mediated signaling, spanning various second messengers, and GR-mediated transcriptional activity, along with cooperativity with other transcription factors influencing the expression of about 10–20% of the human genome (as reviewed in [25]), it is conceivable that there are interactive influences on the physiological effects of AR and GR activation. The primary evidence for interactive influence comes from the therapeutic efficacy of combinational therapy involving β_2_AR agonists as bronchodilators and GR agonists as immunosuppressants. To understand the mechanisms of the collective influence of a combination therapy involving bronchodilators and steroids, Mostafa et al. [126] examined global transcriptome changes induced by individual and combination treatment in airway epithelial cells. This study demonstrated that the action of glucocorticoids agonist budesonide suppressed the transcription of proinflammatory gene expression in cells, while enhancing the transcription of apoptosis, proliferation, differentiation, and other functional processes in cells treated with the β_2_AR-specific agonist formoterol, suggesting that the interactive effects at multiple levels may influence the effectiveness of the therapy. These authors have further reported that long-acting βAR agonists do not necessarily enhance the expression of all glucocorticoid-inducible genes, but have gene-specific effects [127].

The interactive influence of glucocorticoids and catecholamines has also been reported at the level of the neuroendocrine system. Glucocorticoid modulation of pituitary adrenocorticotropic hormone (ACTH) release can alter thymus catecholamine availability through its influence on sympathetic nerve terminals, and change AR gene expression [128]. It has been suggested that dexamethasone-induced GR activation interferes with the trafficking and degradation of the β-arrestin–α_2_cAR complex in human neuroblastoma cells [129]. Long-term use of bronchodilators has been reported to cause β_2_AR desensitization in airway smooth muscle cells and reduction in therapeutic efficacy. It has been shown that GPCR-kinase-mediated phosphorylation of ligand-bound receptors leads to β-arrestin binding, which prohibits further receptor G_as_ coupling and cellular signaling [130]. GR activation normally reduces β-arrestin 2 levels in lung epithelial cells, which is hypothesized to counteract the β-arrestin-2-induced desensitization and, thus, increase the therapeutic efficacy of bronchodilators [92]. This reduction would enable β_2_AR agonists to activate signaling to cause airway smooth muscle relaxation and improve lung function. The activation of β_2_AR has been shown to enhance GR-mediated transactivation through G_bg_ subunits and PI3 kinase [131]. Stimulation of β_2_AR increased cAMP, and led to cross-talk between CREB proteins and GRs [132]. It is likely that these interactive effects might involve common second messengers, leading to changes in downstream signaling events. Thus, interactions between AR signaling and GR-mediated transcriptional changes are likely involved in the therapeutic efficacy of combination treatment using β_2_AR and GR agonists for asthma and COPD. Because ozone exposure was shown to increase both epinephrine and corticosterone, the potential for interactive effects through endogenous AR and GR ligands is also likely. The combination treatment with the β_2_AR agonist clenbuterol and the GR agonist dexamethasone in rats, at therapeutically relevant dose levels, dampened ozone-induced inflammatory responses when compared to the responses induced by clenbuterol alone [6]. Future studies on air pollutants should examine the interactive influence of activating AR subtypes and GRs.

## 9. Air Pollution’s Impact on Circadian Clock Genes, and the Potential Mediating Roles of ARs and GRs

Neuroendocrine pathways that mediate ozone effects are also known to regulate the expression of circadian clock genes and associated physiological changes (reviewed in [133,134]), raising the possibility that circadian mechanisms together with AR and GR signaling might also be involved in air pollution health effects. Exposure to air pollution has been associated with disturbed sleep cycles and obstructive sleep apnea [135]. Cantone et al. [136] recently reported that in acute ischemic stroke patients, PM exposure was linked to changes in methylation of the CLOCK regulated genes. PM exposure in utero has also been shown to disrupt the placental epigenetic signatures of CLOCK genes in women, which may be linked to inflammatory changes [137]. Experimental evidence has begun to emerge linking exposure to air pollutants to changes in the expression of circadian genes, and to downstream signaling events that lead to changes in inflammation and metabolic processes. Exposure to air pollutants in mice has been reported to induce changes in chromatin dynamics by downregulating histone acetylases, causing increased promotor occupancy, altering the expression of genes involved in neuroendocrine-mediated circadian rhythmicity, and subsequent changes in genes regulating brown adipose tissue as well as liver metabolic processes [138]. These processes have been shown to be regulated at the cellular level through ARs and GRs [139], implying that oscillatory changes in the levels of circulating adrenal-derived hormones are linked to changes in neuronally induced diurnal rhythmicity.

**Figure 4:**
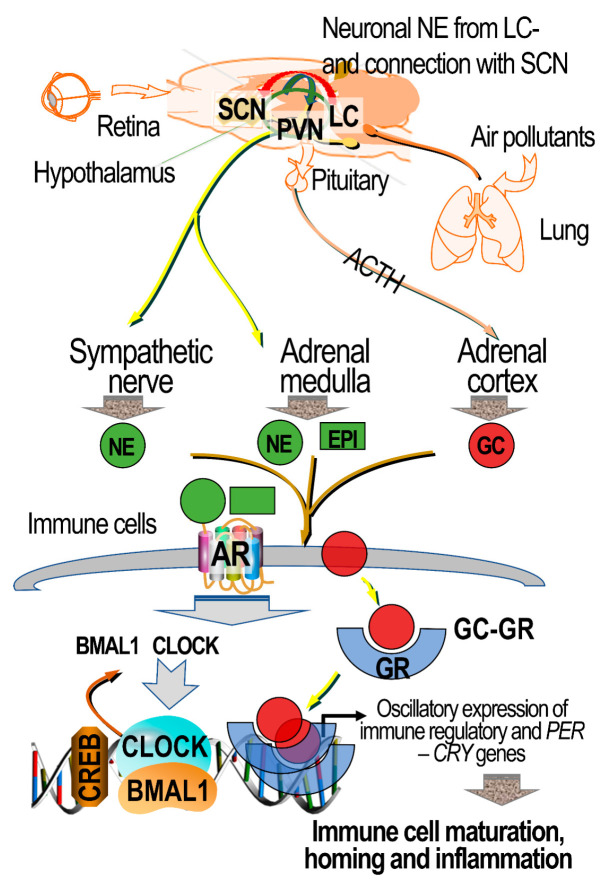
Proposed schematic of how adrenergic and glucocorticoid mechanisms regulate circadian changes and environmental stress signals to direct immune responses with oscillatory patterns. The suprachiasmatic nucleus (SCN), receiving photonic signals from the retina via the retinohypothalamic tract, transmits these to the paraventricular nucleus (PVN) of the hypothalamus, which also integrates other stress signals from afferent autonomic sensory nerves, including those induced by pulmonary encountered air-pollution-induced stress. These signals are integrated in the hypothalamus and relayed to the periphery through: (1) sympathetic nerves, which transmit signals to the peripheral organs by releasing norepinephrine (NE); (2) sympathetic nerves innervating the adrenal medulla and regulating the production and release of epinephrine (EPI) and norepinephrine into circulation; and (3) the hypothalamus–pituitary–adrenal (HPA) axis mediating the pituitary release of adrenocorticotropic hormone (ACTH), and then stimulating glucocorticoid (GC) production by the adrenal cortex. Adrenal glucocorticoids locally regulate the release of medullary hormones. Catecholamines and glucocorticoids released into circulation induce pulsatile cellular physiological changes resulting from stress and circadian rhythms through binding to their receptors—AR and GR subtypes, respectively. Within the central nervous system, the locus coeruleus (LC) produces norepinephrine, which is transmitted across many brain regions, including the SCN, and can regulate circadian changes centrally. Circulating catecholamines and glucocorticoids bind to ARs and GRs in diverse organs and cells, including immune cells, to regulate the expression of the circadian locomotor output cycles kaput (CLOCK) and brain and muscle aryl hydrocarbon receptor nuclear translocator-like 1 (BMAL1) regulated genes. The signaling involves the activation of transcription factors, including the cyclic AMP response element-binding protein (CREB), to modulate the expression of CLOCK and BMAL1-regulated genes. The CLOCK and BMAL1 transcription factors regulate the transcription of genes encoding circadian proteins, such as period circadian proteins (PERs) and cryptochromes (CRY). The rhythmic activation of GRs upon binding to GCs, and their nuclear translocation, can modulate gene expression for inflammatory processes in association with CLOCK and BLAM1 in immune cells that facilitate diurnal changes in maturation and homing of immune cells and inflammation.

Since the SAM and HPA axes coregulate circadian and stress-induced peripheral immune and metabolic homeostasis, it is conceivable that health effects induced by air pollutants through this pathway could impair circadian rhythmicity and associated peripheral changes (Figure 4). Diurnal and stress-induced variations occur not only in metabolic processes, but also in the immune surveillance to assure protection during active period [139,140]. Hypothalamus, the master regulator of environmental cues, receives stress signals from external and internal stressors though autonomic sensory nerves and light signals directly from the suprachiasmatic nucleus (SCN), which is connected to the retina [141]. The hypothalamus integrates the information received to from both sources to generate bodily response through neural and endocrine mechanisms involving the SAM, HPA, and other hormonal axes to direct peripheral immune and metabolic responses via the activation of ARs and GRs [17]. Diurnal oscillations are evolved through complex self-regulatory mechanisms of genes and transcription factors in the brain and other peripheral organs, and work in tandem with adrenergic and glucocorticoid mechanisms to mediate physiological changes to synchronize with changes in activity patterns. The oscillatory pattern in response to diurnal changes programmed by the hypothalamus is communicated to the periphery through sympathetic nerves and the SAM and HPA axes [133,134]. This results in oscillatory changes in the production of norepinephrine at nerve terminals, as well as catecholamines and glucocorticoids within adrenals and blood, which then mediate pulsatile downstream changes in immune cell maturation, egress, and inflammation through ARs and GRs (Figure 4).

Rhythmic variations in catecholamines and glucocorticoid release [142] are regulated by transcriptional regulatory loops involving the circadian locomotor output cycles kaput (CLOCK) and brain and muscle aryl hydrocarbon receptor nuclear translocator-like 1 (BMAL1), which drive period circadian protein (PER) and cryptochrome (CRY) genes by binding to their promoters [143,144]. Brain-derived norepinephrine from the locus coeruleus is presumed to modulate SCN-mediated oscillatory changes [145,146], contributing to circadian changes in sympathetic and HPA-mediated regulation of immune surveillance in the periphery [139,140]. Likewise, GRs also demonstrate oscillatory patterns of diurnal changes [142] through binding to glucocorticoids, and exert cellular effects through the regulation of inflammatory gene expression. Thus, although currently no studies have linked changes in circadian regulatory mechanisms through ARs and GRs following air pollution exposure, it is conceivable that this receptor system is critical in mediating responses to environmental cues, including diurnal variations through light cycle. Given the current evidence that AR and GR functionality is critical in ozone-induced pulmonary and systemic effects, and that exposure to air pollutants is linked to alterations in sleep cycle and genes involved in circadian rhythmicity through epigenetic processes, future air pollution studies should consider a comprehensive assessment of the neuroendocrine stress response system, that includes circadian mechanisms.

## 10. Research Gaps and Opportunities

We demonstrated that ozone, a prototypic oxidant air pollutant, induces its effects via the neuroendocrine-mediated release of epinephrine and corticosterone, which cause cellular effects by interacting with their receptors—ARs and GRs, respectively [5]. Being an oxidant air pollutant, on inhalation, ozone initially interacts with the airway surface lining and alveolar components [80]. Within minutes after an initial encounter with ozone, there is an increase in the sympathetically mediated release of epinephrine, followed by the HPA-mediated release of corticosterone (endogenous ligands for ARs and GRs), prior to increases in cytokine mRNA in the lungs or inflammation [85]. The mechanism by which this initial communication between the lungs and the brain occurs is critical to understanding the role of these receptors in mediating the health effects of air pollution, because, in the absence of these circulating receptor ligands, ozone produces neither lung injury/inflammation nor brain effects [5,7,113]. Although the autonomic sensory innervation and lung–brain neural communication are well studied [147], it is not well understood how the initial irritation induced by air pollutants in the lungs is communicated to the brain in order to induce a neuroendocrine stress response involving ARs and GRs. It has been postulated that bioactive mediators released by the lung cells are responsible for the extrapulmonary and even brain effects of air pollutants [148,149]; however, inflammation takes several hours to occur, but neuroendocrine effects are noted within an hour [85]. Although our studies show that activation of ARs and GRs is required, and that the SAM and HPA axes are stimulated within minutes of ozone exposure, resulting in increased circulating epinephrine and corticosterone, it is important to understand how the initial event induces the neural axes, as well as the role of circulating stress hormones in mediating this event through ARs and GRs.

Due to the diversity of AR and GR subtypes, their involvement in multicellular responses and regulations, and their essential roles in physiological processes, it is difficult to identify one or more specific proteins or genes that will reflect the activity of these receptors. Likewise, assessing one biological response at a time may provide an incomplete understanding of the integrated roles of ARs and GRs in mediating and regulating circadian oscillatory changes and stress. Therefore, in order to clearly delineate the contribution of these receptors, a comprehensive assessment of AR- and GR-regulated processes involving the neuroendocrine system is needed. Experimentally, it is possible to determine the genomic effects of GR activation through transcriptional changes in downstream gene targets; however, the interactive influence of other transcription factors and the downstream effects of GRs are difficult to assess in isolation. Moreover, AR/GPCR signaling is complex, and involves multiple G proteins and second messengers, which are critical in investigating the specific mechanisms. Thus, novel and multipronged approaches together with consideration of temporality are needed in order to identify the precise contributions of ARs and GRs and their coregulation in inducing air pollutant-induced changes. The availability of a wide variety of specific receptor agonists and antagonists for both AR and GR subtypes offers the opportunity to assess the roles of each receptor subtype in cellular effects induced by air pollutants.

Since ARs and GRs are involved in neuroendocrine stress and circadian responses that involve peripheral tissue effects, assessing their contributions provides insights into how air pollutants may affect multiple organ systems, and how the failure of the multifaceted neuroendocrine system to function normally can contribute to chronic diseases. Because ARs and GRs have been implicated in regulation of stress and stress adaptation in the brain [16,17,19,150], it is likely that any malfunction of these receptors because of underlying chronic psychosocial stresses or other conditions, such as altered exposure to the light–dark cycle, will modify how air pollution’s effects are mediated. The evaluation of these receptors in the interactive cellular effects of environmental and psychosocial stressors, as well as circadian rhythmicity, will be critical to understanding individual variability in the health effects of air pollution. Incorporating the roles of ARs and GRs will also have important implications for individuals receiving steroidal and bronchodilator treatments, since they may have exacerbated responses to air pollutants. Thus, future air pollution studies will benefit from assessing the roles of various neuroendocrine hormones, and how their influence on ARs and GRs mediates cellular effects and downstream molecular events in response to stress and diurnal changes.

## Figures and Tables

**Table 1:** Adrenergic (AR) and glucocorticoid (GR) receptor subtypes and their substrate preferences, tissue distribution, and cellular functions. AR and GR subtypes have been well characterized, and are widely manipulated therapeutically. Their wide but selective tissue distribution, efficacy for ligands, and receptor-subtype-specific functionality are critical in maintaining temporal and dynamic changes in biological processes to regulate homeostasis. EPI: epinephrine; NE: norepinephrine; CNS: central nervous system; SNS: sympathetic nervous system.

Receptor Type	Affinity for Endogenous Substrate	Tissue Distribution	Cellular Response	References
AR-α_1_	EPI ≥ NE	Vascular smooth muscle, heart	Vasoconstriction	[28,29,32]
AR-α_2_	EPI ≥ NE	Presynaptic adrenergic and cholinergic nerve terminals (postsynaptic CNS)	Inhibition of transmitter release (SNS outflow reduction)	[29,32]
AR-β_1_	EPI = NE	Heart, kidney (glomerular cells)	Increases heart rate, ventricular muscle contraction, increases renin release	[28,30,32,33]
AR-β_2_	EPI >> NE	Smooth muscle cells (respiratory, vascular, and uterine), respiratory epithelial cells	Increases smooth muscle relaxation, increases glucose in liver, fluid balance, proinflammatory (increases contractility in the heart)	[1,28,32]
AR-β_3_	NE > EPI	Adipose tissue cells, bladder	Increases lipolysis in adipose tissue and relaxes the bladder muscle	[31,32]
GR-α	Endogenous glucocorticoids	All tissue and cell types	Nuclear translocation, activation/inhibition of genes, nongenomic regulation of cellular processes	[25,26]
GR-β	No ligand	All tissues, abundant in neutrophils and epithelial cells	Localized in the nucleus, inhibits GR-alpha activity, involved in glucocorticoid resistance	[25]

**Table 2:** Selected respirable particulate matter (PM) and acrolein studies incorporating the roles of adrenergic receptors (ARs) and glucocorticoid receptors (GRs) and/or their endogenous ligands—catecholamines and glucocorticoids, respectively. Only the data pertaining to ARs and GRs are summarized in the table. * There are a number of cigarette smoke and other studies that have implicated the contribution of ARs and GRs to observed health effects, but only one example is provided. LPS: lipopolysaccharides; PM: respirable particulate matter; NF-κB: nuclear factor kappa B; IL-6: interleukin 6; NO_2_: nitrogen dioxide.

Pollutant Type	Model System	Receptor Subtype	Study Design and Outcome	Reference
Ambient PM	Human trial	Endogenous ligands for ARs and GRs	PM exposure increased cortisol, epinephrine, norepinephrine, and changed glucose and lipid metabolites in serum.	[89]
Ambient NO_2_ (Traffic)	Epidemiology	Endogenous GR ligand	NO_2_ but not PM exposure was associated with increased morning cortisol in plasma.	[90]
Ambient pollutants	Epidemiology	Endogenous AR ligand	Ambient pollution was associated with increases in urine catecholamines.	[91]
Ambient PM	Dog	αARs	Dogs exposed to ambient PM through tracheal tube had increased blood pressure, and this PM effect was inhibited by αAR antagonists.	[88]
Cigarette smoke *	Lung epithelial cell line	β_2_AR-associated second messengers	Suppression of inflammatory cytokine production through β-arrestin signaling was linked to βARs and inhibition of autophagy through AMPK in cigarette-smoke-condensate-exposed cells.	[92]
LPS	Macrophage cell line	β_2_AR and β-arrestin	β_2_AR negatively regulated NF-κB by β-arrestin 2, and through stabilizing the NF-κB/IκB-α complex.	[93]
Ambient PM	Mice in vivo, and human macrophages	β_2_AR and its ligand	PM exposure in mice increased circulating catecholamines and macrophage IL-6 release. In human macrophages, β_2_AR agonists increased—and antagonists decreased—IL-6 production.	[8]
Acrolein	Rat	Endogenous ligands for ARs and GRs	Acrolein inhalation increased corticosterone and epinephrine in Wistar and diabetic Goto–Kakizaki rats, which were associated with nasal injury and inflammation.	[94]
Ambient PM	*Adra2b*-transgenic mice	α2AR	Concentrated PM exposure increased blood pressure, and anxiety-like behavior, which was associated with upregulation of inflammatory genes in the brains of *Adra2b*-transgenic mice, overexpressing *α_2_bAR*.	[95]
Diesel exhaust	Endothelial cells	βARs	In endothelial cells, diesel exhaust extract increased inflammatory cytokines’ release, and this effect was inhibited by βARs and calcium channel inhibitors in an extract-specific manner.	[3]
Ambient PM	Rat microvessels, *ex vivo*	αARs	Microvessels isolated from PM-exposed rats had inhibited endothelium-dependent arteriolar dilation. αARs inhibited PM effects.	[96]
Ambient air pollution	Humans and mice	Endogenous ligands for GRs	Exposure to air pollution was associated with increased plasma cortisol in humans and corticosterone in mice. In mice, PM increased hippocampal inflammation and inhibited GR expression.	[97]
Metal mixture	Mouse macrophage cell line	GR activation	GR activity was inhibited by selected metals, as indicated by reporter luciferase assay.	[98]
Ambient PM	Rat	GRs	Increased expression of genes regulated by activation of GRs in multiple tissues, including lung.	[99]

**Table 3:** Selected experimental studies involving ozone and the roles of adrenergic receptor (AR) and glucocorticoid receptor (GR) subtypes and/or their endogenous ligands—catecholamines and glucocorticoids, respectively. This table is not meant to be a comprehensive list of all experimental studies that mention ARs and/or GRs; rather, ozone studies focused on the lungs and addressing the roles of ARs and GRs and their endogenous ligands are listed.

Model System	Receptor Subtype	Study Results	References
Human	Endogenous ligands for GRs	In a clinical study, ozone exposure increased plasma levels of cortisol, which was associated with increased lipid metabolites	[110]
Rat	Endogenous ligands for ARs	Epinephrine level increased in rats immediately after ozone exposure, and this was associated with lung injury inflammation and lymphopenia.	[14,105]
Rat	Endogenous ligand manipulation for ARs and GRs	Adrenal demedullation diminished circulating epinephrine, and total adrenalectomy diminished both epinephrine and corticosterone. This was associated with inhibition of ozone-induced lung injury, inflammation, lymphopenia, and lung expression of genes involved in AR and GR signaling, acute-phase response, hypoxia, and inflammation.	[4,113,114]
Rat	β_2_AR and GR agonists, individually or in combination	Pretreatment of rats with β_2_AR agonists exacerbated ozone-induced lung injury and inflammation. GR agonists, but not β_2_AR agonists, exacerbated ozone-induced lymphopenia. Combination treatment exacerbated both lymphopenia and lung effects, including gene expression of inflammatory markers and GR-responsive targets, in both sham and adrenalectomized rats.	[4,6,15]
Rat	βAR and GR antagonists	βAR antagonists suppressed ozone-induced lung vascular leakage and neutrophilia, while GR antagonists reversed lymphopenia but not lung neutrophilia. The combination of both antagonists inhibited all ozone-induced effects.	[5]
Rat	Endogenous ligands of ARs and GRs in brain effects	Depletion of circulating endogenous ligands, epinephrine, and corticosterone by adrenalectomy inhibited ozone-induced changes in gene expression within the brainstem and hypothalamus. This was associated with the reversal of ozone-induced decreases in circulating prolactin, luteinizing hormone, and thyroid-stimulating hormone.	[7]
Rat	Endogenous ligands for ARs and GRs	Over a 4-h period of ozone exposure, circulating epinephrine and corticosterone increased. These increases were followed by the depletion of circulating granulocytes, M1 monocytes, B and T lymphocytes, and lung expression of GR-regulated genes. Only small changes occurred in circulating cytokines.	[85]
Rat	Endogenous ligands for GRs	Ozone exposure increased corticosterone in lung lavage fluid and inhibited alveolar macrophage cytokine production. The stress-sensitive Fischer 344 strain exhibited greater effects than those of stress-resistant Lewis rats. Inhibiting corticosterone production increased inflammatory cytokine expression in macrophages.	[115]
